# Frameless linac-based radiosurgery for benign intracranial tumors treated with HyperArc: analysis of tumor control and toxicity

**DOI:** 10.1007/s11060-025-05291-8

**Published:** 2025-10-21

**Authors:** Whitney S. Hotsinpiller, Evan M. Thomas, Ian Tsekouras, Richard A. Popple, Markus Bredel, Christopher D. Willey, Barton L. Guthrie, James M. Markert, Kristen O. Riley, John B. Fiveash, Drexell Hunter Boggs

**Affiliations:** 1 Department of Radiation Oncology, The University of Alabama at Birmingham, Birmingham, AL USA; 2 Renaissance Institute of Precision Oncology & Radiosurgery, Winter Park, FL USA; 3 Department of Radiation Oncology, The University of Miami, Miami, FL USA; 4 Department of Neurosurgery, The University of Alabama at Birmingham, Birmingham, AL USA; 5 University of Alabama at Birmingham, Hazelrig-Salter Radiation Oncology Center (176F) RM 1240B, Birmingham, AL USA

**Keywords:** Radiosurgery, HyperArc, Meningioma, Benign, Tumor, Radiation

## Abstract

**Purpose:**

HyperArc™ (HA) automates both planning and delivery of single-isocenter VMAT radiosurgery (SRS) and was designed for complex multi-metastasis cases. The clinical effectiveness of treating benign intracranial tumors (BIT) with HA is unknown. We collected data on treatment planning, delivery, and clinical outcomes of BIT managed with SRS since HA deployment.

**Methods:**

Patients received SRS using HA from 2017 to 2021 at a single institution. Prescription dose was normalized to ≥ 99% of gross tumor volume without additional expansion. Treatments were delivered on Varian Edge linear accelerator with 10MV flattening-filter free beam at up to 2400 MU/min with high-definition multi-leaf collimator. Post-treatment imaging, toxicities, and outcomes were assessed.

**Results:**

198 BIT targets (min = 0.1 cc, max = 58.9 cc) were treated. A large variety (*n* = 8) of BIT were treated with the most common pathologies being meningiomas (130), pituitary adenomas (30), and acoustic schwannomas (23). Nearly half (45.1%) were treated in a single fraction (12–22 Gy) versus 54.9% with fractionated SRS (24–35 Gy). Mean RTOG CI and Paddick GI were 1.12 and 3.31, respectively. A majority (74%) were treated with 3 arcs with mean treatments lasting 10.5 min. Mean FU was 2.6 years. 20 of 198 (10.1%) tumors progressed with mean time to failure being 2.1 years. Tumor progression occurred in 10.1%, mostly in recurent WHO II meningiomas. Of those that progressed, 19 were meningiomas with 17 being WHO grade 2 with prior surgery. Grade 3 + CNS toxicity was reported in 4.3% of patients, with 98.9 and 99.5% hearing and visual preservation, respectively.

**Conclusions:**

Without any planning target volume expansion, HA efficiently delivers high-quality plans with sharp dose fall-off for a wide variety of BITs. Early clinical outcomes and toxicity are consistent with historical controls.

## Introduction

GammaKnife and CyberKnife modalities are the traditional standard for benign intracranial tumor radiosurgery due to their conformality and minimal dose to normal brain [1–3]. However, these systems use point-and-shoot geometries, which are inherently less efficient than modulated, arc-based delivery enable by multi-leaf collimators (MLCs). Since the introduction of treating tumors with volumetric modulated arc therapy (VMAT) in 2008, efficiency and quality of radiation planning has improved for many disease sites including the central nervous system [4]. Radiosurgery, which incorporates VMAT-techniques, flattening filter-free beams, and high dose rates, has become more prevalent for radiating intracranial pathologies due to the ability to increase dose per fraction as well as sharpen dose fall-off, sparing healthy brain tissue [5]. With increasing utilization of radiosurgery, an automated single-isocenter VMAT planning and delivery system for linac-based radiosurgery was developed by Varian Medical Systems called HyperArc™ (HA, Varian Medical System Inc., Palo Alto, CA, USA). HyperArc™ completed development in 2017 and was intended for complex multi-metastasis cases for which it generates high-quality, rapidly-deliverable plans [6–8]. The effectiveness of treating benign intracranial tumors with HyperArc without additional PTV margin is unknown.

Radiosurgery remains a standard of care for benign intracranial tumors that do not undergo resection. With much longer life expectancy than that of multiple malignant brain metastases and slower tumor growth, it is imperative to create high-quality, safe radiosurgery plans. Long and short-term outcomes of treating benign intracranial tumors with manually-planned and delivered radiosurgery provide optimal local control with minimal toxicity [9]. Under an IRB-approved prospective registry, we investigate treatment planning, delivery, tumor control and toxicity of patients with benign intracranial tumors treated with HyperArc™.

## Methods

This project was approved by the IRB. HyperArc™ was implemented in October 2017 at a single institution. Shortly after implementation, the first few patients with benign intracranial tumors were treated. Patients included received radiosurgery using HyperArc™ for benign intracranial tumors between May 10th 2018 and October 14th 2021. Patients with less than three-month follow-up with brain imaging were excluded. Treatment of multiple targets during one treatment was allowed. Prior treatment with radiation and/or surgery was also allowed. Grade III meningiomas were excluded.

The methods used for this study have been previously described [8]. To summarize, all patients were simulated supine wearing an Encompass™ open mask immobilization device required for HyperArc™ automation. Computed tomography (CT) simulation with 1-milimeter slice thickness was performed from 2 centimeters above the cranium to the second cervical vertebral body inferiorly and thus encompassed the entirety of the cranium. Intravenous contrast was provided unless clinically contraindicated.

For a large majority of cases, at least 1or more magnetic resonance (MR) data sets were registered to planning CT for more accurate target delineation. MR registration was not used if MR was clinically contraindicated or in very select cases if tumor clearly visible on planning CT. The most common sequence registered to the planning CT was a thin sliced (1 mm) T1-weighted sagittal post-contrast MR data set. Using Eclipse (version 15), high resolution target volume contours were created and reviewed in collaboration with both a radiation oncologist and neurosurgeon. Treatment volume was calculated within the Eclipse system based on contoured gross tumor volume (GTV) as a high-resolution object. For all targets, full prescription dose was normalized to ≥ 99% of GTV without additional planning target volume (PTV) expansion. Also contoured were clinically relevant organs at risk (OARs) such as brain, brainstem, normal brain (brain subtracting GTV), optic structures, and cochlea. Fractionation and dose to tumor varied based on type of tumor and tumor size.

HyperArc™ created the automated plans using at least two, but up to four arcs (partial or complete) at fixed, predetermined angles. All plans were calculated using a 10 MV flattening filter-free beam (2400 MU/min maximum dose rate) on a Varian Edge linear accelerator equipped with a high-definition (2.5 mm) multi-leaf collimator. Plan evaluation involved review of total target volume, Paddick conformity index, Paddick gradient index, maximum dose, and clinically relevant dose to relevant organs at risk [10–12]. The Paddick conformity index and gradient index were calculated for both single-fraction and multi-fraction radiosurgery plans. Tumors were categorized based on location and dose maximum on plan evaluation as being within or without close proximity to critical structures. To be considered within close proximity to brainstem, the tumor had to be within 1.5 mm and have a dose maximum of at least 8 Gy. To be considered within close proximity to cochlea or optic structures (nerve or chiasm), the tumor had to be within 1.5 mm and have a dose maximum of at least 3 Gy. After plan approval and prior to treatment, quality assurance was completed with radiochromic film which was digitized and calibrated to assess alignment, mean dose, and gamma index [13]. Pre-treatment patient specific quality assurance (PSQA) was done by measuring the dose in a coronal plane centered on the target volume. For multiple lesions, two measurements were done with the plane centered on the the smallest and largest target volumes. Prior to March 2020, radiochromic film was used [8]. Subsequently, a two-dimensional diode array was used for PSQA [22]. Radiochromic film measurements demonstrated high geometric accuracy for HyperArc plans, which had a median registration offset from the planned dose distribution of 0.32 mm [8]. Machine quality assurance followed the guidelines given in the report of the American Association of Physicists in Medicine Task Group 142 report [25]. All machine parameters met the SRS tolerance values recommended by the TG-142 report. For example, the median imaging and treatment coordinate coincidence over the period of this study was 0.2 mm.

Treatment delivery began with patients initially positioned prior to treatment using orthogonal kV images registered to the treatment-planning CT followed by a cone beam CT and secondary registration. The treatment table (PerfectPitch™) allowed 6 degrees of freedom corrections for both registrations. Intrafraction motion was monitored continuously with optical surface imaging [23]. Phantom measured surface image walkouts at each couch position verified most translational errors were less than 0.5 mm with outlier maximum shifts < 1 mm [24]. Treatment timestamps were obtained from the Eclipse database and used to define beam-on and total treatment delivery time. Total treatment delivery time included patient setup, cone beam CT, image registration, and beam on-time.

Post-treatment, patients were followed in either or both outpatient neurosurgery and radiation oncology clinics with routine follow-up imaging. Local tumor control, toxicity, and further interventions were recorded at each visit. Tumor progression was defined as greater than 25% increase in tumor size or pathologic confirmation of tumor recurrence. Definite and probable radiation induced toxicities were defined using the Common Terminology Criteria for Adverse Events (CTCAE), version 5 [14], with significant toxicity having a grade of three or greater.

Descriptive statistics related to SRS treatment, pathology, and prior therapy determined at the time of or prior to treatment were analyzed. Post-treatment imaging, toxicities, and standard pathology-specific were analyzed during follow-up visits.

## Results

In total, 198 benign intracranial targets were treated with 186 radiosurgeries using HyperArc™ in 183 patients. A large majority (62.8%, *n* = 115) of patients were female and the mean age was 59.0 ± 14.0 years ranging from 15 to 89 years. Nearly half (49.7%, *n* = 91) of patients had recurrent, residual, or persistent disease with 49.1% (*n* = 90) having prior surgery and 14.7% (*n* = 27) having prior radiation. A few patients (3.3% *n* = 6) had a prior diagnosis of neurofibromatosis type II (Table 1, *Section A*). The most common pathologies for each radiosurgery were meningioma (65.5%, *n* = 130), pituitary adenoma (15.2%, *n* = 30), acoustic schwannoma (11.6%, *n* = 23), and paraganglioma (4.5%, *n* = 9). Other benign tumors (3.2%) included craniopharyngioma (*n* = 3), angiofibroma (*n* = 1), hemangioblastoma (*n* = 1), and pineal tumor (*n* = 1). All patients with more than one target had multiple meningiomas. No patient had more than two targets.

**Table 1:** (A) patient demographics and tumor descriptors. (B) summary of treatment planning measures including target size, dose, fractionaction, planning objectives, and proximity to critical organs. *Only single fraction SRS (C) summary of treatment delivery time and technique. (D) summary of follow-up, tumor control, and toxicity for benign intracranial tumors treated with hyperarc planned and delivered radiosurgery

**A: Patient Demographics**						
	**Number**, ***n***	**Percentage**, **%**	**Mean**, ***n***	**SD**, ***n***		
Patients	183					
Radiosurgeries	186					
Targets	198					
Female	115	61.8%				
Male	71	38.2%				
Age			59.0	14.0		
Recurrent/Residual Tumor	91	49.7%				
Prior Surgery	90	49.1%				
Prior Radiation	27	14.7%				
Neurofibromatosis Type 2	6	3.3%				
**B: Treatment Planning**						
	**Number**,** n**	**Percentage**,** %**	**Mean**,** n**	**SD**,** n**	**Min**,** n**	**Max**,** n**
Treatment Volume (ccs)			7.6	4.3	0.1	58.9
Single Fraction SRS (Gy)	84	45.1%			12	22
Three-Fraction SRS (Gy)	4	2.2%			21	27
Five-Fraction SRS (Gy)	98	52.7%			25	30
Total Dose (Gy)			20.7	7.2	12	30
Dose per Fraction (Gy/fx)			8.9	4.1	5	22
RTOG Conformity Index			1.1	0.1		
Gradient Index			3.3	0.6		
Dose Maximum (%)			133.9	8.9	105.9	164
Brain Receiving 12 Gy* (ccs)	84		2.5	2.1	0.1	10.1
Near Brainstem, Max Dose (Gy)	76	38.4%	18.5	6.8	8.4	31.7
Near Optics, Max Dose (Gy)	74	37.3%	12.1	7.4	3	26.5
Near Cochlea, Max Dose (Gy)	59	29.7%	12.3	7.1	3	33.3
**C: Treatment Delivery**						
	**Number**,** n**	**Percentage**,** %**	**Mean**,** n**	**SD**,** n**	**Min**,** n**	**Max**,** n**
2 Arcs	47	25.3				
3 Arcs	139	74.7				
Total Treatment Time (mins)			10.5	2.2	7.2	30.2
Beam-on Time (mins)			2.2	0.4	1.4	4.3
**D: Follow-up**,** Control**,** and Toxicity**						
	**Number**,** n**	**Percentage**,** %**	**Mean**,** n**	**SD**,** n**	**Min**,** n**	**Max**,** n**
Follow-up (days)			962.1	422.8	106	1876
Tumor Progression	20	10.1				
Tumor Decrease in Size	35	17.7				
Tumor Remain Stable	131	66.1				
Tumor Increase Treatment Effect	12	6.1				
CTCAE Grade 3 or higher	8	4.3				
Required Avastin	9	4.9				
Subjective Vision Decline	13	7.1				
Vision Preservation	182	99.5				
Subjective Hearing Decline	6	3.2				
Hearing Preservation	181	98.9				

**Fig. 1:**
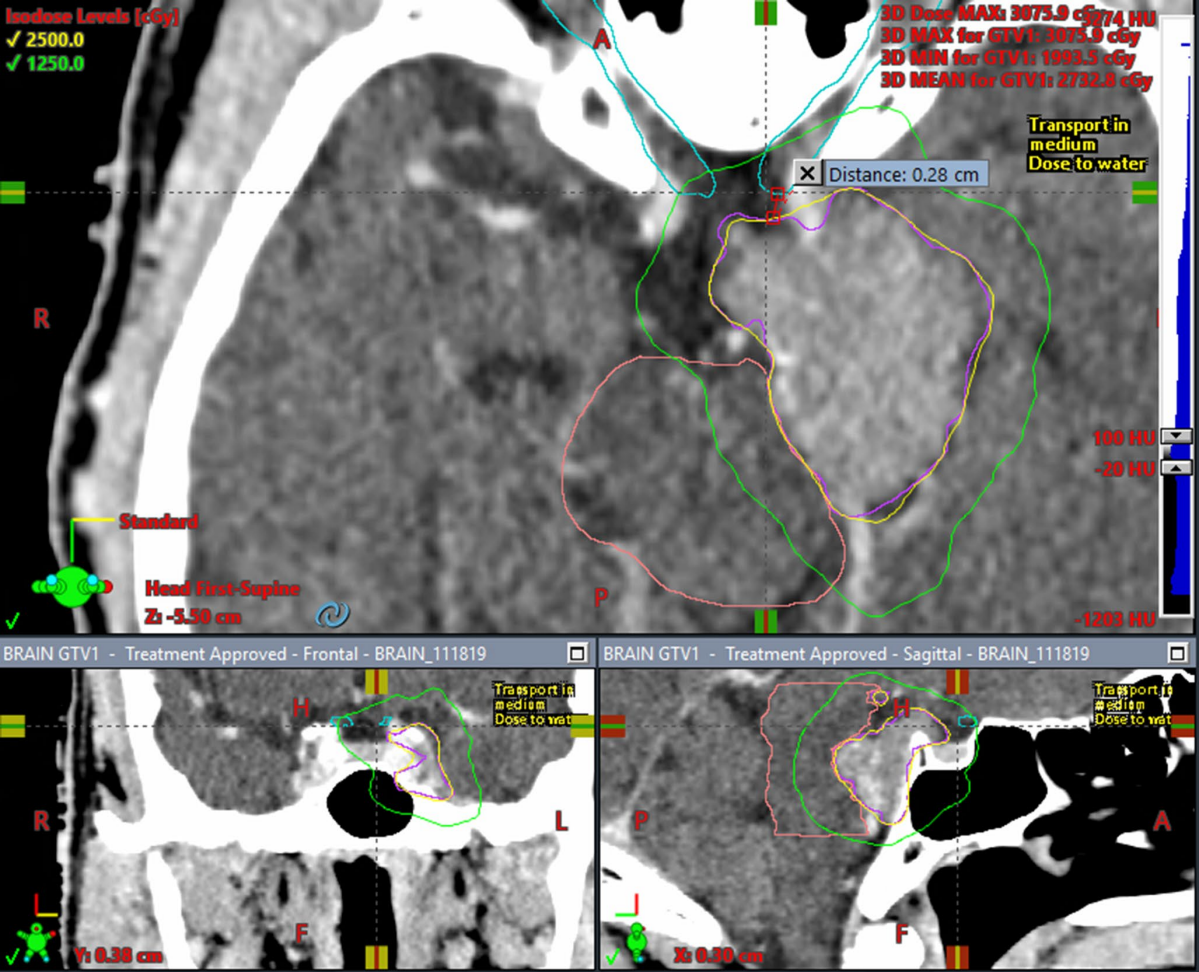
Three-plane (superior – axial; inferior left – coronal; inferior right – sagittal) view of HyperArc plan for a meningioma (magenta contour – GTV with no PTV margin) that is abutting the brainstem (coral contour) and 0.28 cm from the left optic nerve (cyan contour) being treated to a dose of 2500 cGy as demonstrated by the 100% yellow isodose line. The 50% isodse line (1250 cGy) is green

**Fig. 2:**
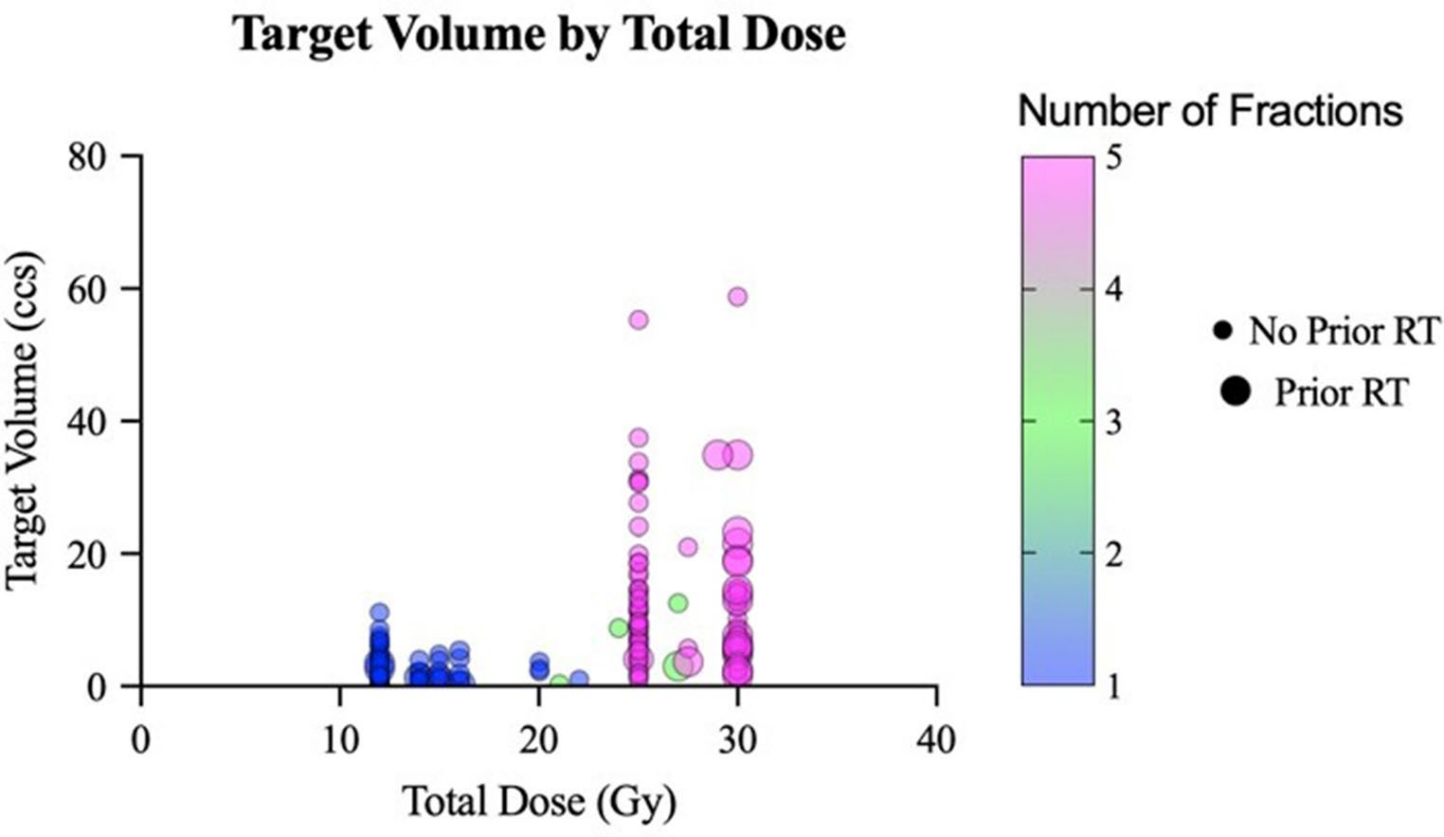
Dose by target volume is plotted. Larger volume tumors were more likely to receive 5-fraction radiosurgery (25–30 Gy) as opposed to smaller volume tumors that received single fraction (12–22 Gy). 3-fraction radiosurgery (21–27 Gy) was used more frequently for small-medium voume tumors that were abutting critical OARs. Treatments where patient had prior RT were more likely to receive 5-fraction radiosurgery

**Fig. 3:**
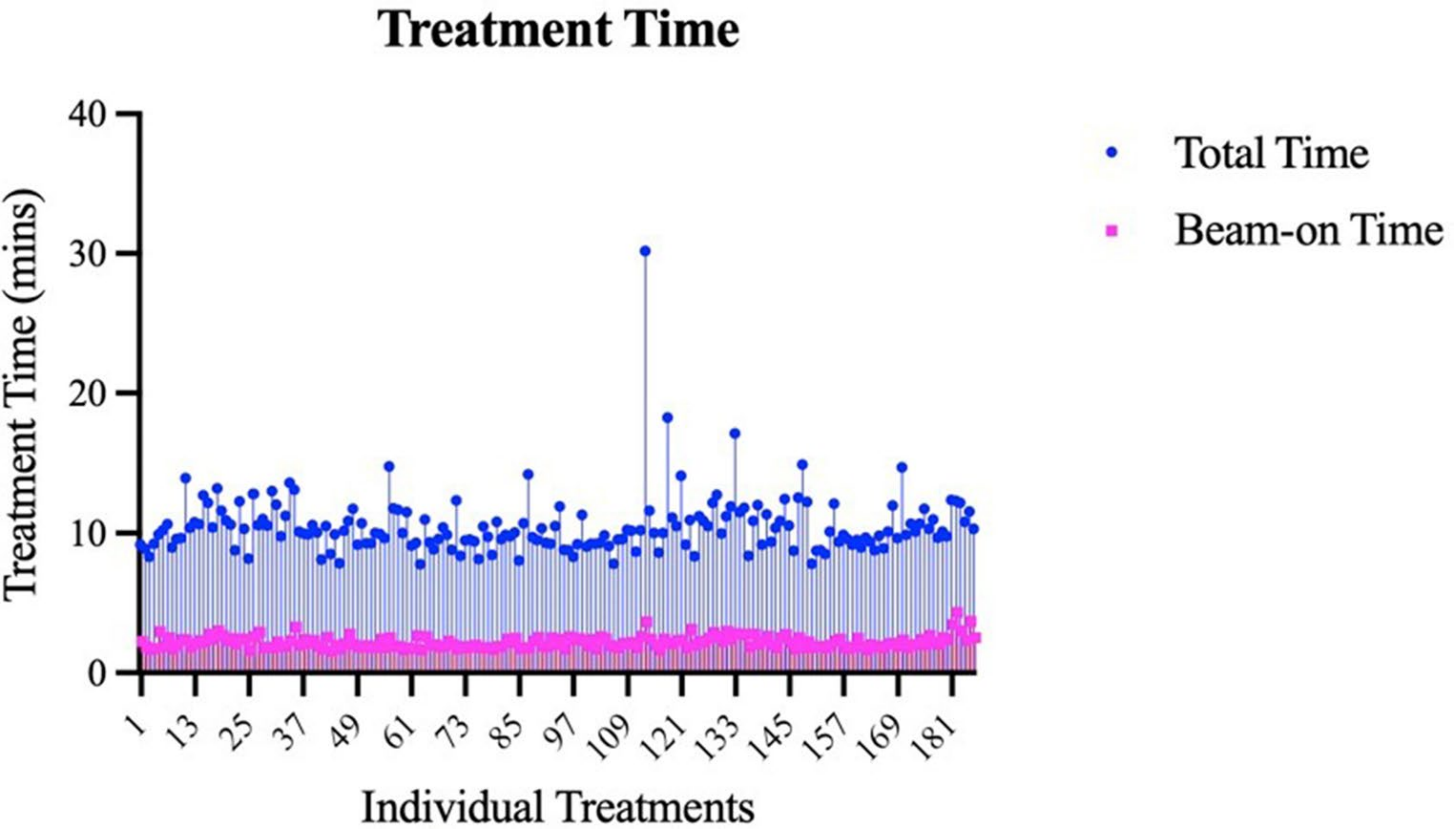
Bar graph demonstrating beam-on time and total treatment time for each frameless linac-based radiosurgery case. The mean beam-on time and total treatment time were 2.2 min and 10.5 min, respectively. One outlier case required multiple re-alignment CBCTs due to movement and thus total treatment time was 30.2 min

After contouring, target volumes were calculated within Eclipse and ranged from 0.1 to 58.9 cubic centimeters (ccs) with a median of 4.25 ccs and mean of 7.56 ccs. Nearly half (45.1%, *n* = 84) were treated in a single fraction (12–22 Gy), 54.9% were treated with fractionated SRS (21–35 Gy). The mean dose overall was 20.7 ± 7.2 Gy with a mean dose per fraction (fx) of 8.9 ± 4.1 Gy/fx. The mean RTOG CI and Paddick GI were 1.12 ± 0.1 and 3.31 ± 0.5, respectively. The mean dose maximum was 133.9 ± 8.9% ranging from 105.9 to 164.0%. For single fraction SRS, the mean volume of normal brain receiving 12 Gy was 2.49 ccs. The percentage of targets defined to be within close proximity of the brainstem, optics, and cochlea were 38.4% (*n* = 76), 37.3% (*n* = 74), and 29.7% (*n* = 59), respectively (Table 1, *Section B*). An example of a plan treating a tumor abutting the brainstem and near the optics can be seen in in Figure 1. Fractionation and dose to tumor varied based on type of tumor, tumor size, prior RT, and proximity to critical OARs (Figure 2). A majority (74%) were treated with 3 arcs with mean total treatment and beam-on time lasting 10.5 ± 2.2 and 2.2 ± 0.4 min, respectively (Table 1, *Section C;* Figure 3).

**Fig. 4:**
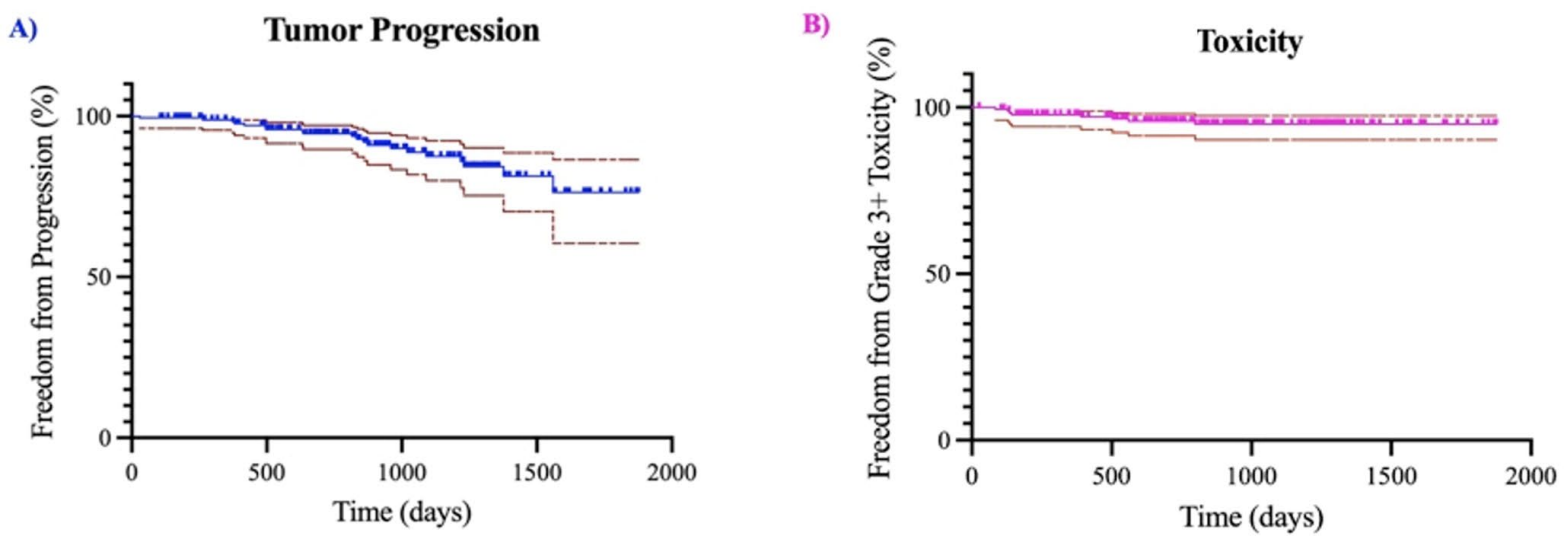
Kaplan-Meier survival curves demonstrating freedom from progression per patient (**a**) and freedom from significant toxicity (CTCAE grade 3+) per patient (**b**) over time. The maroon lines demonstrate the 95% confidence intervals

The mean follow-up was 962 ± 423 days (2.6 ± 1.2 years). Progression occurred for 20 targets (10.1%) on follow-up imaging with a mean time to failure of 778 days (2.13 years). Some tumors decreased in size (17.7%, *n* = 35), some slightly increased favoring treatment effect (6.1%, *n* = 12), while the rest remained stable (66.1%, *n* = 131). Of those that progressed, 19 were meningiomas of which 17 were WHO grade II with prior surgical and/or radiation management. Significant CNS toxicity was reported in 4.3% of patients, most of whom had cerebral edema requiring medical (*n* = 7) and/or surgical intervention (*n* = 3). Only 2 of the 8 patients with significant CNS toxicity had prior RT. Kaplan-meier curves plotting tumor progression (section A) and significant toxicity (section B) over time are displayed in Figure 4. Of these patients, all received dexamethasone while only some required bevacizumab. A total of 9 patients were treated with bevacizumab due to concerns of radiation necrosis. If patients reported decreased vision but had functional vision without major deterioration of quality of life, this was reported as preserved vision. Of those with tumors within close proximity of the optics without severe vision loss prior to SRS, 17.5% (*n* = 13) of patients subjectively reported decreased vision after treatment, although 98.6% (*n* = 73) self-reported preserved vision in the treated eye(s). Of those with tumors within close proximity of the cochlea without severe hearing loss prior to SRS, 10.1% (*n* = 6) of patients subjectively reported decreased hearing after treatment, although 96.7% (*n* = 57) self-reported preserved hearing in the treated ear (Table 1, *Section D*). For the total population, hearing and vision preservation was 98.9 and 99.5%, respectively. Of the 8 patients who received bevacizumab prophylactically due to prior RT, none had significant radiation related toxicity. Eight patients in this cohort died during follow-up of which five (62.5%) died a tumor-related death all of which were high grade meningiomas.

Given the majority of targets (*n* = 130, radiosurgeries *n* = 118, patients *n* = 115) were meningiomas, subset analysis of meningiomas alone revealed 54.6% (*n* = 71) of targets were radiographically diagnosed whereas WHO grade I and II meningioma targets were diagnosed in 12.3% (*n* = 16), 33.1% (*n* = 43), respectively. Nearly half (40.7%, *n* = 53) of patients had recurrent, residual, or persistent disease. Few (*n* = 6, 5.2%) patients had Neurofibromatosis type II. Only 37.2% (*n* = 44) of radiosurgeries were treated in a single fraction, with most having been treated with 5-fraction SRS (60.1%). Nineteen meningiomas (14.6%) progressed after treatment with WHO grades of I (*n* = 1) and II (*n* = 17). Of the meningiomas that progressed, one had a history of NF2 and was radiographically diagnosed. Most (73%) had CTCAE toxicity of zero or one, whereas 9.8% had significant toxicity.

## Discussion

This study reports the largest known cohort of benign intracranial tumors treated using HyperArc™, a modern, automated VMAT-based stereotactic radiosurgery system. HyperArc was designed as an automated and highly efficient version of single isocenter multiple target VMAT radiosurgery. Applications in other forms of radiosurgery including benign tumors and AVMs remain a question compared to more established forms of radiosurgery. The current study is particularly relevant in that no PTV margin was utilized and that HyperArc SRS can be performed on a multi-purpose linac rather than a dedicated radiosurgery device. Historically, treatments for these tumors have relied on Gamma Knife and CyberKnife due to their high conformality and minimal dose to surrounding brain tissue. As demonstrated, HyperArc™ produces highly conformal plans with low dose spill, while offering significantly more efficient treatment times compared to the point-and-shoot systems used by Gamma Knife and CyberKnife [1–3]. Although HyperArc^™^ is based on VMAT, the main advantage of HyperArc^™^ is simplication and partial automation of the treatment planning process including omission of ring-based tuning structures in the cost function. Other linac systems including those using dynamic conformal arcs (e.g. BrainLab Elements) may be less conformal due to lack of beam modulation and computer optimization of beam fluence, but have potential advantages of less beam modulation and facilitated patient specific quality assurance. Comparative dosimetric studies demonstrate lower GI/V12 Gy isodose spill of Hyperarc™ when compared to CyberKnife and improved CI and GI for single target cases when compared with Gamma Knife [20, 21]. With comparable dosimetry, local control, and toxicity—paired with superior efficiency—HyperArc™ is a safe and effective alternative to established radiosurgical modalities for treating benign intracranial tumors.

Although HyperArc™ was originally developed for complex, multi-metastatic radiosurgery, it has proven highly effective for treating benign intracranial tumors, producing high-quality, rapidly deliverable plans with sharp dose fall-off [8, 15]. Unlike metastatic brain disease, benign intracranial tumors typically involve a single target, and patients often live for decades following appropriate treatment. In our cohort, over half of the patients had undergone prior radiation therapy or surgery, emphasizing the importance of minimizing dose to surrounding organs at risk (OARs), such as normal brain tissue. Many benign tumors are located adjacent to critical structures—for example, the optic apparatus in pituitary adenomas and the cochlea or brainstem in acoustic schwannomas. In our study, approximately 40% of tumors were in close proximity to the brainstem. HyperArc™ produced exceptional treatment plans, with a mean RTOG conformity index (CI) of 1.12 and gradient index (GI) of 3.31. Notably, the standard deviation for CI was only 0.1, demonstrating robust and precise optimization across a wide range of target volumes (0.1 to 58.9 cc). As defined by Paddick [11] the gradient index is the ratio of volume of half the prescription isodose to the prescription isodose volume. This ratio is impacted by changing conformity of the treatment plan such that more conformal plans will have a smaller prescription isodose volume. VMAT plans typically have a high degree of conformity [ref. 8] which will result in a numerically worse gradient for the same volume receiving half of the prescription. For single-fraction SRS, the mean volume of normal brain receiving 12 Gy (V12) was just 2.5 cc, underscoring the system’s ability to deliver highly conformal treatment with excellent normal tissue sparing.

Another compelling argument for the use of HyperArc™ in the radiosurgical treatment of benign intracranial tumors is its automation of the treatment delivery process. Utilizing selected arc placements with a single isocenter and a frameless Encompass™ mask, HyperArc™ enables standardized, efficient treatment delivery. In our study, most treatments required only 2–3 arcs, with an average delivery time of 10.5 min. With dose rates up to 2400 MU/min, HyperArc™ achieves treatment speeds 4 to 8 times faster than Gamma Knife (320 MU/min) or CyberKnife (1000 MU/min), while maintaining high conformality and minimal dose spill. As illustrated in Figure 3, treatment times were consistently efficient, averaging approximately 10 min in total. While manually planned and delivered radiosurgery has shown success for benign tumors, HyperArc™ provides several key advantages, including improved planning efficiency, reduced treatment time, and superior dosimetric precision with steep dose gradients. These benefits are especially pronounced in cases involving multiple tumors, such as in many of the NF2 patients in our cohort, where automation and efficiency become increasingly critical.

With treating patients nearly since deployment of HyperArc™ in late 2017 at our institution, maximum length of follow-up for this database is up to 5.1 years. With a mean follow-up of 2.6 years and prospective data collection, acute toxicity was accurately assessed as well as some late toxicity. Over 75% of patients had only CTCAE grade 0 or 1 toxicity with no treatment-related deaths. With nearly 40% of tumors being within 1.5 mm of the optic apparatus and with a maximum dose to optic structures of at least 3 Gy, incredibly 99.5% of patients preserved vision function. The 17.5% reported subjective vision loss rate included both transient and permanent vision loss without definitive relation to SRS. With nearly 30% of tumors being with 1.5 mm of the cochlea and with a maximum dose to cochlea of at least 3 Gy, 98.5% of patients preserved hearing function. Thus, hyperarc appears to generate safe plans to tumors nearly abutting or completely abutting critical OARs.

Long-term control rates for pituitary adenomas, WHO grade I meningiomas, and acoustic schwannomas are approximately 90% [9]. Given only 2 of these tumors recurred during our mean follow-up of 2.6 years, the recurrence rates appear congruent with historical controls even without use of PTV margins. Not surprisingly, the vast majority of tumors that recurred in our study were recurrent WHO grade II status-post surgery and/or radiation therapy. Treating WHO grade II meningiomas with stereotactic radiosurgery is less defined in literature as many facilities continue to treat with a prolonged fractionated course [16]. Studies predict local control to be approximately 80–90% at 1 year and 30–50% at 5 years [17, 18]. Meningiomas tend to be more aggressive in the recurrent setting [19]. Nearly half of our patients with meningioma presented with recurrent, persistent, or residual disease prior to treatment with stereotactic radiosurgery. Of the WHO grade II meningiomas treated with HyperArc SRS, our local control rate at approximately 2.6 years was 85.4% and appears to be congruent with historic controls although comparison is difficult due to limited studies and WHO grade migration.

This is the first report on utilizing linac basesd SRS plans with 0 mm PTV margins automated by HyperArc™ to treat benign intracranial tumors. The strengths of this study lie in its large patient cohort and its comprehensive approach to assessing both treatment outcomes and potential toxicities. Importantly, this series includes a wide range of benign tumors pathologies. Additionally, the study’s prospective registry design ensures systematic data collection for each follow-up to accurately code tumor control and patient toxicity. Although this is the largest cohort of benign intracranial tumors treated with HyperArc™ planned radiosurgery, the study is limited by being single-arm and single-institution. Other limitations include short follow-up time (late toxicity and control) and lack of data to show overall planning time using HyperArc™. Further limitations included hearing and vision being patient reported outcomes without formal audiometry and/or visual acuity testing. Future studies should consider adding audiometric and ophthalmologic assessments both prior to radiosurgery and on follow-up given the close proximity of many of these benign intracranial tumors to cranial nerves.

## Conclusions

In conclusion, this study presents the largest known cohort of benign intracranial tumors treated with HyperArc™, demonstrating its effectiveness, efficiency, and safety in delivering high-quality, automated VMAT-based stereotactic radiosurgery with 0 mm PTV margins. HyperArc™ consistently produced highly conformal plans with sharp dose gradients, excellent normal tissue sparing, and rapid delivery—averaging just over 10 min per treatment—while maintaining local control and toxicity outcomes comparable to historical standards set by Gamma Knife and CyberKnife. Notably, the system performed exceptionally well in treating tumors adjacent to critical structures such as the optic apparatus and cochlea, preserving vision and hearing in over 98% of cases. With a mean follow-up of 2.6 years, acute and some late toxicities were accurately assessed, and local control rates remained consistent with or better than published outcomes, even in challenging cases like recurrent WHO grade II meningiomas. Long term followup of outcomes and toxicity for this patient population is needed given the indolent nature of this disease. Use of HyperArc™ in treatment of benign intracranial tumors will automate treatment planning and delivery without compromising plan quality or clinical outcomes.

## Data Availability

The datasets generated during and/or analysed during the current study are available from the corresponding author on reasonable request.
